# Pathological Process of Prompt Connection between Host and Donor Tissue Vasculature Causing Rapid Perfusion of the Engineered Donor Tissue after Transplantation

**DOI:** 10.3390/ijms19124102

**Published:** 2018-12-18

**Authors:** Sachiko Sekiya, Shunichi Morikawa, Taichi Ezaki, Tatsuya Shimizu

**Affiliations:** 1 Institute of Advanced Biomedical Engineering and Science, Tokyo Women’s Medical University, Tokyo 162-8666, Japan; shimizu.tatsuya@twmu.ac.jp; 2 Department of Anatomy and Developmental Biology, Tokyo Women’s Medical University, Tokyo 162-8666, Japan;ezakit@twmu.ac.jp (T.E.)

**Keywords:** tissue engineering, transplantation, host–donor, vasculature connection

## Abstract

The shortage of donors for transplantation therapy is a serious issue worldwide. Tissue engineering is considered a potential solution to this problem. Connection and perfusion in engineered tissues after transplantation is vital for the survival of the transplanted tissue, especially for tissues requiring blood perfusion to receive nutrients, such as the heart. A myocardial cell sheet containing an endothelial cell network structure was fabricated in vitro using cell sheet technology. Transplantation of the three-dimensional (3D) tissue by layering myocardial sheets could ameliorate ischemic heart disease in a rat model. The endothelial cell network in the 3D tissue was able to rapidly connect to host vasculature and begin perfusion within 24 h after transplantation. In this review, we compare and discuss the engineered tissue–host vasculature connection process between tissue engineered constructs with hydrogels and cell sheets by histological analysis. This review provides information that may be useful for further improvements of in vivo engineered tissue vascularization techniques.

## 1. Introduction

Organ transplantation is used as an effective alternative approach to treat diseases that do not respond to the usual therapies. However, the number of available donors always falls short of the number of potential recipients awaiting transplants. Even when patients are able to find donors, many issues, such as age, pre-existing physical conditions, and cold ischemia time, may affect the survival of the transplanted organ [1,2].

In order to resolve issues related to donor shortages, researchers are investigating new biotechnological tools for fabricating donor tissue [3,4,5]. Blood perfusion starting time is closely associated with survival and treatment efficacy, even in engineered organs or tissues. Specifically, myocardial tissues, fabricated with myocardial cell sheets including endothelial cell (EC) network structures, require prompt perfusion after transplantation [6]. These tissues perfuse much more rapidly compared to other engineered tissues [7].

In this review, we will discuss the factors that influence the connection and beginning of blood perfusion in engineered tissues after transplantation. Utilizing the histological assessment of myocardial tissue engineered via cell sheet technology before and after transplantation, we can better understand how the connection process of host–donor vasculature in engineered tissues causes prompt perfusion in vivo. Because of their rapid connection and blood perfusion, layered myocardial cell sheets of over 1-mm thickness could be fabricated by polysurgery in the backs of rats. The knowledge of the connecting process, which includes pericyte function, may be useful for understanding vascularization, which occurs in not only engineered tissue transplantation, but also wound healing and tumor growth in vivo.

## 2. Factors Affecting Host–Donor Vasculature Connection Causing Donor Tissue Perfusion

**Figure 1:**
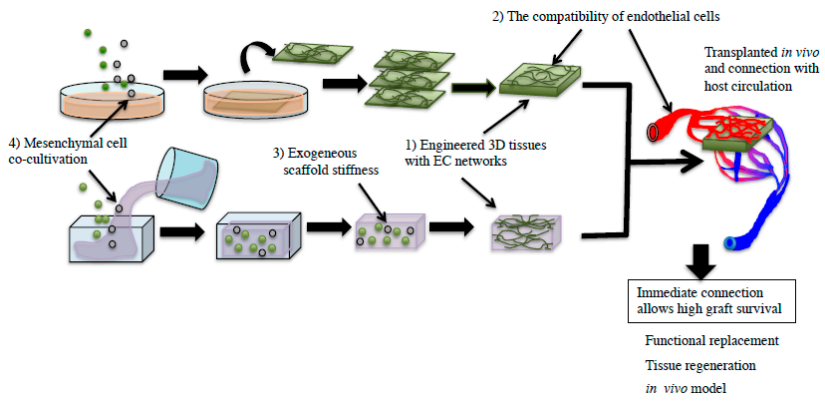
Factors associated with connection and perfusion of transplanted tissues. Using tissue engineering technology, 3D tissues were formed and transplanted. Factors affecting host–donor vasculature connection and perfusion are: (1) Engineered 3D tissues with endothelial cell (EC) networks, (2) compatibility of endothelial cells, (3) exogenous scaffold stiffness, and (4) mesenchymal cell co-cultivation. The transparent circles were indicated as mesenchymal cells. The green circles were indicated as ECs.

Several factors affect the initiation of the blood perfusion period in engineered donor tissues following in vivo transplantation. It is considered that (1) the EC network structure is important for initiating blood perfusion periods in donor tissues after transplantation [8]. The EC network in donor tissues was highly efficient in fabricating functional vasculature in vivo after transplantation [9,10]. Furthermore, (2) the compatibility of ECs between the donor and recipient is important. It was reported that the human umbilical vein endothelial cell (HUVEC) network delayed circulation in host mice compared to mouse EC networks [7]. The next factor (3) is tissue containing an exogenous scaffold gel. The gel stiffness influenced the migration of ECs and vascular endothelial growth factor (VEGF) secreted from smooth muscle cells (SMCs) [11,12]. Moreover, individual tissue stiffness alters the vasculature of the tissue in vivo. (4) Peri-vascular mesenchymal cells, neural/glial antigen 2 (NG2)-positive pericytes, α-smooth muscle actin (SMA)-positive SMCs, and mesenchymal stromal cells (MSCs) especially affected perfusion of transplanted tissues co-cultivated with them [13,14]. Thus, understanding the effects of these factors may enable the establishment of controllable connections between the engineered tissue–host vasculature, causing prompt perfusion of donor tissues after transplantation (Figure 1). These controllable connections may be useful in future regenerative medicine endeavors, not only for the heart, but also for kidney and liver regeneration.

## 3. Establishment of Blood Perfusion in Engineered Tissues after Transplantation

### 3.1. Tissue Engineering Technology Including Cell Sheet Technology

“Tissue engineering” represents the field of implantable tissue fabrication, using cells, for clinical therapy. Biodegradable scaffolds, extracellular matrix (ECM), and decellularized scaffolds have been utilized for engineering 3D tissues [15]. Cell sheet technology is one such tissue engineering technology used for the fabrication of two-dimensional (2D) cell sheets. The 2D cell sheet can be obtained with layer-by-layer technology by exogenous ECM coating and centrifugation [16], but cell sheet technology also enables fabrication of 2D cell sheets using temperature-responsive culture dishes [17]. Only a reduced culture temperature of confluent cultured cells on temperature-responsive culture dishes enables 2D cell sheet harvest with intact cell–cell interaction and adhesive factors without proteolytic treatment. Therefore, cell sheets showed higher survival after transplantation, and 3D tissues could be fabricated by layering 2D sheets. This advantage may improve cell therapy for clinical applications [18].

### 3.2. Blood Perfusion throughout Engineered Tissue after Transplantation

Previously, many types of engineered tissues with EC networks have been transplanted in vivo. They could perfuse blood through neovasculature originating from the EC networks. ECs embedded in hydrogels were reported to be able to start perfusion within 6 days after transplantation [7]. The collagen-hydrogel was co-cultured with ECs and mesenchymal cells for 24 h before transplantation. The host mouse ECs gradually invaded the grafted tissue. Host and grafted ECs were connected at the border area between host and graft tissue, and facilitated blood perfusion. The hydrogel containing an EC network was not capable of complete perfusion until 28 days after transplantation. In collagen gels, ECs could not form a tubular structure themselves, despite the addition of VEGF [19]. However, phorbol myristate acetate (PMA) or fibroblast co-culture resulted in large-diameter (over 25 µm) lumens in the collagen gel [20]. According to these results, large EC lumens in collagen gel required only 6 days to start perfusion. Moreover, the EC structure was retained for 6 days until starting blood perfusion in vivo.

**Figure 2:**
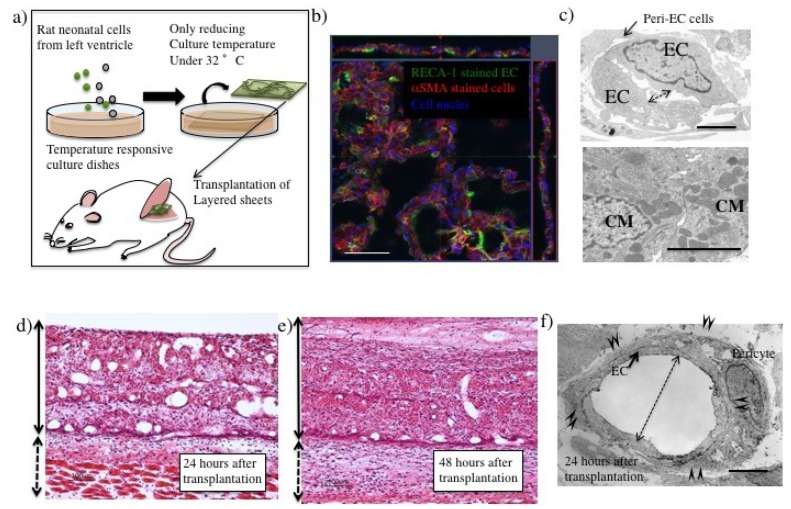
The pathological assessments of donor myocardial cell sheets before and after transplantation. (**a**) Procedures for myocardial cell sheet transplantation into rats. (**b**) Image of immunofluorescence staining of myocardial cell sheet cultured for 4 days in vitro by confocal microscopy. EC (rat endothelial cell antigen (RECA)-1: green), peri-EC cells (αSMA: red), nuclei (Hoechst 33258). (**c**) Electron microscopic analysis of ECs and cardiomyocytes (CMs) in myocardial cell sheets cultured for 4 days in vitro. Peri-EC cells (arrow) already attached to ECs forming narrow lumens (two direction arrow). Lower image shows cardiomyocytes in the cell sheets. (**d**) Hematoxylin and eosin (H.E.) staining of a transplanted myocardial cell sheet 24 h after transplantation and (**e**) 48 h after transplantation. Side solid lines with two direction arrows indicate transplanted cell sheet area, and short dashed lines of two direction arrows indicate host tissue. (**f**) Vasculature lumens in transplanted myocardial cell sheets. Pericytes connected to ECs tightly (arrow-heads) to form large lumens (two directional arrow). Scale bar: (**b**,**d**,**e**) about 100 µm, (**c**,**f**) 3.3 µm. (The scale bar was added with estimation from original data.)

Alternately, the myocardial cell sheet with EC networks fabricated from a primary culture of rat neonatal left ventricular tissues showed vascular-like structures with ECs and pericytes or smooth muscle cells in vitro following cultivation for 4 days (Figure 2b). EC networks contained narrow lumens (estimated to be under 1 µm in diameter) surrounded by thick ECs in the myocardial cell sheet (Figure 2c). After transplantation on the fascia in the backs of rats however, vascular ECs showed a thinner shape (Figure 2f) than that before transplantation. Compared to EC networks in collagen gel, the alteration of EC structure in tissues engineered with cell sheet technology was unique.

Moreover, vasculature with a large diameter was observed in the transplanted tissue at 24 h (Figure 2d). It was also reported that layering EC- and fibroblast-containing cell sheets increased lumens by the segmentation of existing large diameter lumens in vitro [21]. Thus, vasculature remodeling occurred rapidly in the layered cell sheets via the process of segmentation of large lumens. This resembled the intussusceptive angiogenesis reported previously [22]. VEGF concentration was associated with the process of vascular segmentation after myoblast suspension injection. A high dose (121 ng VEGF/10^6^ cells) of VEGF caused non-sprouting angiogenesis [23,24]. VEGF was also released from neonatal rat cardiomyocytes [25], causing intussusceptive angiogenesis. Alternately, the segmentation process might result in the alteration of EC polarity in the cell sheets. EC networks in a cell sheet form an apical-basolateral polarity in vitro. However, this polarity is altered by layering with another sheet or gel. EC sheets showed a network structure immediately after being layered with fibroblast cell sheets or gel stamps [26]. As EC proliferation is not necessary for the segmentation process of myocardial cell sheets, blood perfusion of the donor tissue was more rapid than that in the sprouting process of angiogenesis.

### 3.3. The Process of Host–Donor Vasculature Connection after Transplantation

When the 3D tissue, engineered in vitro, is transplanted in vivo without surgical vascular anastomosis, the host vasculature and EC network in the 3D-engineered tissue should be connected to each other for survival of implants. In this process, host-graft ECs should be connected after overcoming the surrounding pericytes and SMCs. This process requires degradation and re-creation of the surrounding ECM, and dynamic migration of ECs and surrounding cells.

**Figure 3:**
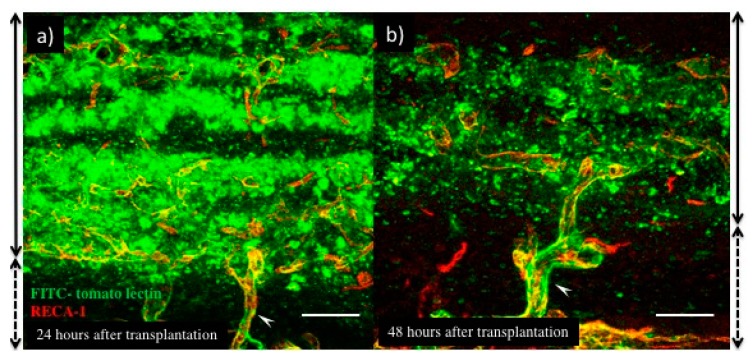
Fluorescein isothiocyanate (FITC)-tomato lectin perfused in host rats 24 h (**a**) or 48 h (**b**) after myocardial cell sheet transplantation. Green indicates FITC-tomato lectin and red indicates RECA-1 positive ECs. Side solid lines with two directional arrows indicate the transplanted cell sheets area, and short dashed lines with two directional arrows indicate host tissues. White arrows were indicated as a host-graft connection vasculature. Scale bar: (**a**,**b**), about 100 µm. (The scale bar was added with estimation from original data.)

**Figure 4:**
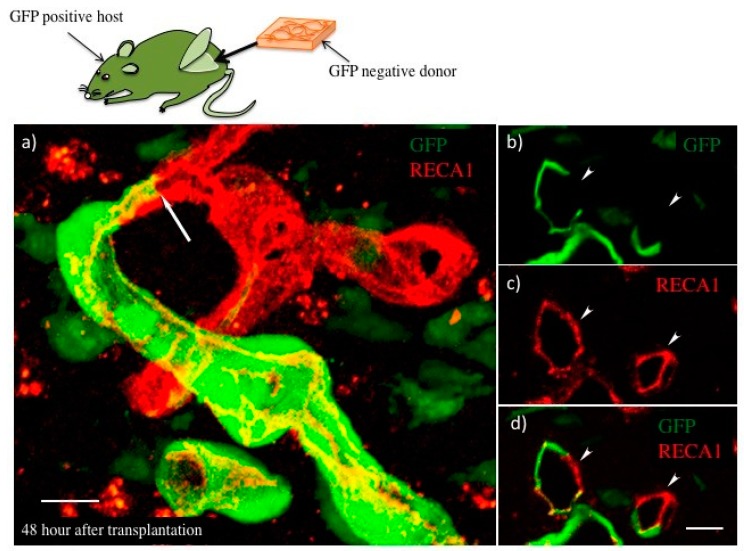
Donor myocardial cell sheets transplanted into GFP+ host rats. (**a**) Host–donor vasculature connecting point shown by confocal microscopy. Green indicates GFP+ cells, and red indicates RECA-1+ ECs. Arrow indicates host–donor EC connecting border. (**b**–**d**) show the section of connecting points. (**b**) GFP+ host originated vascular image and (**c**) RECA-1+ total vascular image. (**d**) A merged image of (**b**) and (**c**). Arrow-heads indicate GFP-vasculature originating from donor. Scale bar: (**a**,**d**), about 10 µm. The scale bar was added with estimation from original data.

**Figure 5:**
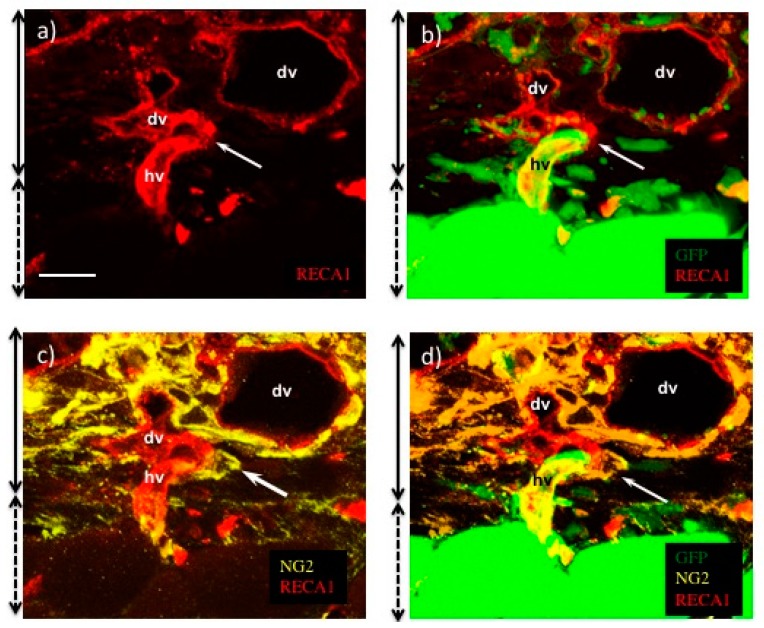
Immunofluorescence staining of the border areas of myocardial cell sheets transplanted in donor and GFP+ host rats. (**a**) An image of RECA-1+ ECs. (**b**) A merged image of Host GFP + cells and RECA-1 + ECs. (**c**) A merged image of RECA-1 + ECs and neural/glial antigen 2 (NG2) + pericytes. (**d**) A merged image of (**b**) and (**c**). dv = donor vasculature, hv = host vasculature, and arrow indicates the connecting point of dv and hv. Side solid lines with two directional arrows indicate the transplanted cell sheets area, and short dashed lines with two directional arrows indicate host tissues. Scale bar: (**a**), about 20 µm. The scale bar was added with estimation from original data.

**Figure 6:**
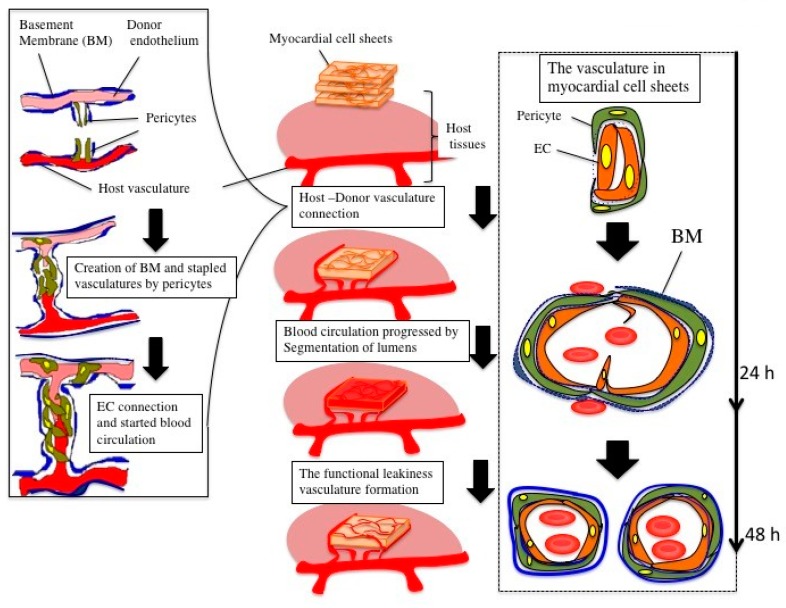
Hypothesis regarding connection and circulation process following engineered myocardial tissue transplantation into rats.

In the case of transplanted myocardial tissues, the number and lumen diameter of the vasculature was decreased at 48 h after transplantation (Figure 2e), and the leaking perfused fluorescein isothiocyanate (FITC)-tomato lectin also decreased (Figure 3b) compared to that at 24 h after transplantation (Figure 2d and Figure 3a). Thus, a remodeling of vasculature progressed in the transplanted tissue, and permanent vasculature remained in the donor tissue. The connection and starting circulation of immature donor vasculature with the host at 24 h after transplantation was a very unusual, prompt process. We also detected connected green fluorescent protein-positive (GFP+) host vasculature to GFP negative (GFP−) donor vasculature at peripheral donor tissues within 24 h after transplantation of the GFP− donor into the GFP+ host rat (Figure 4). The connection point was already covered in Neural/glial antigen 2-positive (NG2+) peri-vascular cells from either the host or donor (Figure 5). There is a possibility that both host and donor peri-vascular cells navigated from host endothelium to donor endothelium to form connections in a staple-like manner (Figure 6). NG2+ (Platelet-derived growth factor receptor-β positive) PDGFβ+ pericytes led to a proliferation of ECs via secretion of VEGF [20] at the wound healing site. This indicated that peri-vascular cells from the host and the donor migrated to the border area of the transplantation and connected with each other. Then, VEGF secreted by peri-vascular cells may induce ECs to connect points of perfusion. At 24 h after transplantation, a massive leakage of perfused lectin was observed in the donor tissue. However, few leaks were observed at the host-donor border connection area (around arrow-head in Figure 3a) at the connection site of host–donor ECs (Figure 3). Thus, vasculature was stabilized promptly following connection, compared to the leaking vasculature in the wound healing assay. Leaking vasculature at 3 days of wound healing was stabilized from 5 to 10 days following wounding [27]. The reason for the prompt connection may be associated with highly potent peri-vascular cells in the donor tissues. Because they were primarily cultured from neonatal rat tissues, they were immature, but possessed a high potential for angiogenesis. The leakiness at 24 h suggested that the donor vasculature was still immature at the time, and then became rapidly functional until 1 day after connection with host circulation. Conversely, tumor pericytes exhibited abnormal shapes [28] and were associated with the leakiness of tumor blood vessels. The leaky vessels were related to tumor growth and metastasis. In fact, platelet-derived growth factor receptor (PDGFR) β inhibition decreased lymphoma growth via depletion of pericytes [29]. Thus, peri-vascular cells, including pericytes, allow prompt implanted tissue perfusion and long-lasting vasculature formation.

## 4. Conclusions

According to our pathological analysis, donor myocardial cell sheets including EC networks and potent pericytes could connect to host vasculature causing rapid donor tissue perfusion within 24 h after transplantation. The leading ECs from host–donor pericytes may cause the prompt connection. Moreover, the segmentation of large-diameter lumens could be associated with the rapid perfusion of host blood throughout donor tissues by 24 h after transplantation. Importantly, the rapidly formed immature donor vasculature could be functional and without leaks until 48 h after transplantation. The prompt host-donor tissue vasculature connection, donor tissue perfusion, and donor vasculature maturation may be related to highly potent pericytes of neonatal donor tissues. The highly potent pericytes may cause such a rapid connection in the engineered tissue with the hydrogel. However, there is no clear pericyte marker, because the same marker is expressed in mesenchymal stem cells, fibroblasts, and macrophages. A better understanding of the phenotypes and functions of these cells may be useful for advancing regenerative medicine or anti-tumor therapy. Cardiac tissues have been created in vitro with perfusion culture systems. In the future, functional renal tissues and hepatic tissues will also be created in vitro. Furthermore, we hope this knowledge will be utilized for in vitro organ fabrication.

## 5. Materials and Methods

### 5.1. Preparation of Myocardial Cell Sheet

Myocardial cells were isolated from the ventricles of 1-day-old GFP-positive (GFP+) or –negative (GFP−) SD neonatal rats (Japan SLC, Inc., Shizuoka, Japan), as reported previously [30]. Primary cultured myocardial cells were cultured at a density of 2.4 × 10^6^ cells per dish on 35-mm temperature-responsive culture dishes (CellSeed, Inc., Tokyo, Japan) in a cell culture incubator at 37 °C with 5% CO_2_. After 4 days, myocardial cells on the dishes were transferred to another cell culture incubator at 20 °C and 5% CO_2_ for 1 h, to harvest the cultured myocardial cells as intact sheets from the temperature-responsive culture dishes. The fabrication method for myocardial tissues, by triple layering myocardial cell sheets, was also reported previously [30].

### 5.2. Transplantation of Fabricated Tissues into Rats

All procedures were conducted in accordance with the guidelines of the Institute of Laboratory Animals at Tokyo Women’s Medical University. The engineered myocardial sheets were transplanted using the same protocol previously reported [30]. Briefly, EGFP-positive or -negative male SD transgenic rats (4–5 weeks old) were anesthetized with intramuscular injection of ketamine (87 mg/kg) plus xylazine (13 mg/kg). Triple-layered myocardial cell sheets were harvested with a sterile polypropylene support sheet and transplanted into dorsal subcutaneous epifascial tissues. The transplanted myocardial tissue was covered with 0.5-mm-thick silicone membranes (Unique Medical Co., Tokyo, Japan) to prevent adhesion to the tissue under the skin, and the incision was finally sutured.

### 5.3. Vessel Permeability Analysis (FITC-Conjugated Tomato Lectin Perfusion)

At 24 and 48 h after transplantation, recipient rats under anesthesia were injected with 200 µL (800 µg/mL) FITC-conjugated tomato lectin (Vector Laboratories, Burlingame, CA, USA) into the femoral vein to enable visualization of blood-perfusable vessels. After 10-min circulation of injected lectin, 400 mL of 2% paraformaldehyde (PFA) in 0.01 M phosphate-buffered saline (PBS, pH 7.4) was administered at a pressure of 120 mmHg.

### 5.4. Tissue Preparation for Histological Analysis

After perfusion and fixation, the transplanted sheets and surrounding recipient tissues were harvested, trimmed, and fixed with PFA overnight at 4 °C. Thereafter, the fixed tissue was washed with PBS and immersed sequentially in PBS containing 10%, 15%, and 30% sucrose at 4 °C. Subsequently, they were embedded in Tissue-Tek OCT compound (Sakura Finetek, Torrance, CA, USA), snap-frozen in liquid nitrogen, and kept at –80 °C until cryo-slices were made.

### 5.5. Immunohistochemistry

Cryo-sections were incubated with 4% Block Ace (KAC Co., Ltd., Kyoto, Japan) and then with primary antibodies in 1% bovine serum albumin (BSA) (Sigma Aldrich, St. Louise, MO, USA) at 4 °C overnight (Primary antibodies were: Mouse monoclonal anti-RECA-1 (MCA970GA, Bio-Rad Laboratories, Inc., Hercules, CA, USA), rabbit polyclonal anti-NG2 (Chemicon, Merck KgaA, Hessen, Germany), and mouse monoclonal anti-αSMA (1A4, DAKO, Agilent Technologies, Inc., Santa Clara, CA, USA). After incubation, they were washed with PBS and incubated with secondary antibodies (Jackson Immuno Research Laboratories Inc., PA) for 3 h at room temperature. Immunostained sections were examined by confocal microscopy (LSM, Carl Zeiss, Baden-Württemberg, Germany).

### 5.6. Transmission Electron Microscopy

Anesthetized rats were perfused with 4% PFA and 2.5% glutaraldehyde in 0.1 M sodium cacodylate buffer. The transplanted tissues were harvested, trimmed, and immersed in the same fixative solution for 2 h at 4 °C. For the myocardial cell sheets, sheets were soaked in the same fixation solution at 4 °C. Specimens were treated with 1% osmium tetroxide for 2 h at 4 °C, and then with saturated uranyl acetate for 3 h at room temperature. Thereafter, the specimens were dehydrated in a graded series of ethanol and embedded in epoxy resin. Semi-thin sections, 0.5 µm in thickness, were made from the resin-embedded specimens and counterstained with toluidine blue for light microscopy. After the semi-thin sections, ultrathin sections (70 nm in thickness) were made, counterstained with saturated uranyl acetate followed by lead citrate, and observed under a Hitachi H-7000 electron microscope (Hitachi High-Technologies Co., Tokyo, Japan).

## Abbreviations

VEGF	vascular endothelial growth factor
NG2	neural/glial antigen 2
ECM	extracellular matrix
GFP	green fluorescent protein
PDGFR	platelet-derived growth factor receptor
FITC	fluorescein isothiocyanate
RECA-1	rat endothelial cell antigen-1
NG2+ PDGFRβ+	NG2 positive and PDGF-β positive
